# From gene to protein—experimental and clinical studies of ACE2 in blood pressure control and arterial hypertension

**DOI:** 10.3389/fphys.2014.00227

**Published:** 2014-06-24

**Authors:** Sheila K. Patel, Elena Velkoska, Melanie Freeman, Bryan Wai, Terase F. Lancefield, Louise M. Burrell

**Affiliations:** ^1^Department of Medicine, Austin Health, University of MelbourneHeidelberg, VIC, Australia; ^2^Department of Cardiology, Austin Health, University of MelbourneHeidelberg, VIC, Australia; ^3^Department of Cardiology, The Northern Hospital, University of MelbourneEpping, VIC, Australia

**Keywords:** renin angiotensin system, angiotensin converting enzyme, angiotensin converting enzyme 2, blood pressure, hypertension

## Abstract

Hypertension is a major risk factor for stroke, coronary events, heart and renal failure, and the renin-angiotensin system (RAS) plays a major role in its pathogenesis. Within the RAS, angiotensin converting enzyme (ACE) converts angiotensin (Ang) I into the vasoconstrictor Ang II. An “alternate” arm of the RAS now exists in which ACE2 counterbalances the effects of the classic RAS through degradation of Ang II, and generation of the vasodilator Ang 1-7. ACE2 is highly expressed in the heart, blood vessels, and kidney. The catalytically active ectodomain of ACE2 undergoes shedding, resulting in ACE2 in the circulation. The *ACE2* gene maps to a quantitative trait locus on the X chromosome in three strains of genetically hypertensive rats, suggesting that ACE2 may be a candidate gene for hypertension. It is hypothesized that disruption of tissue ACE/ACE2 balance results in changes in blood pressure, with increased ACE2 expression protecting against increased blood pressure, and ACE2 deficiency contributing to hypertension. Experimental hypertension studies have measured ACE2 in either the heart or kidney and/or plasma, and have reported that deletion or inhibition of ACE2 leads to hypertension, whilst enhancing ACE2 protects against the development of hypertension, hence increasing ACE2 may be a therapeutic option for the management of high blood pressure in man. There have been relatively few studies of ACE2, either at the gene or the circulating level in patients with hypertension. Plasma ACE2 activity is low in healthy subjects, but elevated in patients with cardiovascular risk factors or cardiovascular disease. Genetic studies have investigated *ACE2* gene polymorphisms with either hypertension or blood pressure, and have produced largely inconsistent findings. This review discusses the evidence regarding ACE2 in experimental hypertension models and the association between circulating ACE2 activity and ACE2 polymorphisms with blood pressure and arterial hypertension in man.

## Introduction

**Figure 1:**
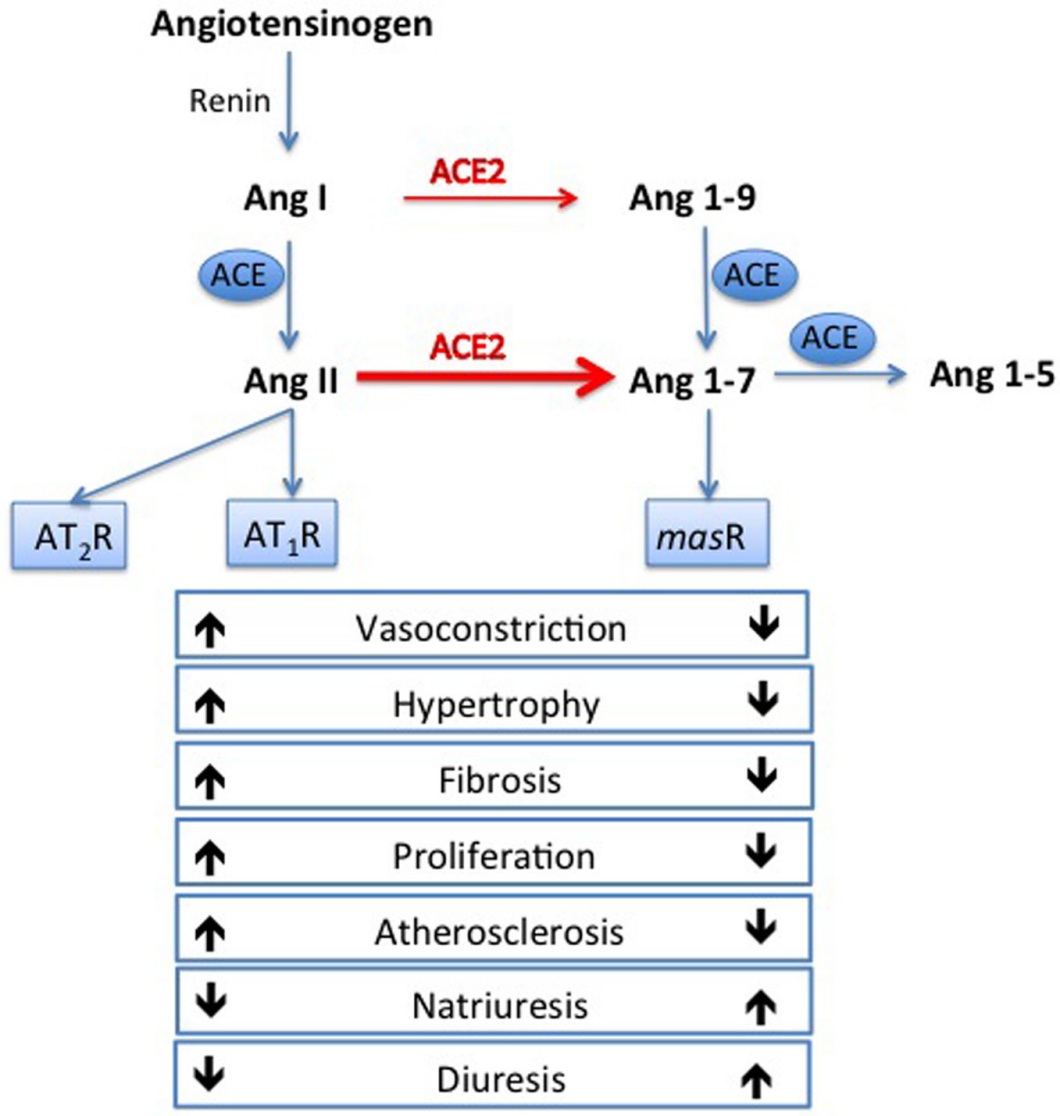
**Opposing arms of the renin angiotensin system**. Angiotensin converting enzyme (ACE) converts angiotensin (Ang) I to Ang II, which exerts its deleterious effects via the angiotensin type 1 receptor (AT_1_R). Ang II can also act on the angiotensin type 2 receptor (AT_2_R) which can counteract AT_1_R mediated effects. In the opposing arm, ACE2 degrades Ang II to Ang 1-7, which is thought to exert beneficial effect via the *mas* receptor.

Cardiovascular disease (CVD) is the leading cause of death and disability worldwide (World Health Organization, 2011), and hypertension is the most common risk factor for CVD (Kearney et al., 2005). The renin angiotensin system (RAS) plays a major role in the pathogenesis of hypertension, which is a risk factor for coronary events, stroke, kidney failure, and heart failure (Johnston, 1994; Dzau, 2005). Within the RAS, angiotensin converting enzyme (ACE) converts angiotensin (Ang) I into the vasoconstrictor Ang II, which mediates its effects via the angiotensin type 1 receptor (AT_1_R). Ang II has actions to raise blood pressure through vasoconstriction and salt and water retention (Figure 1). In experimental models, stimulation of angiotensin II type 2 receptor (AT_2_R) may counteract AT_1_R mediated effects either directly or by modulating AT_1_R signaling (Lemarie and Schiffrin, 2010; McCarthy et al., 2013) In man, therapeutic strategies that block RAS activation using ACE inhibitors or Ang II receptor blockers are first line therapy in hypertension and have beneficial effects on morbidity and mortality.

The discovery of angiotensin converting enzyme 2 (ACE2) (Donoghue et al., 2000; Tipnis et al., 2000) more than a decade ago has led to the concept of an “alternate” arm of the RAS. ACE2 has now been identified in many tissues including blood vessels (Zulli et al., 2006, 2008; Sluimer et al., 2008), heart (Donoghue et al., 2000; Burrell et al., 2005; Ohtsuki et al., 2010), kidney (Tikellis et al., 2003; Mizuiri et al., 2008; Reich et al., 2008), liver (Paizis et al., 2005), and brain (Doobay et al., 2007). The ACE2 amino acid sequence shares approximately 40% homology with the N-terminal catalytic domain of ACE, and a hydrophobic region near the C-terminus likely to serve as a membrane anchor. Like ACE, ACE2 is a type 1 membrane protein with the catalytic domain on the extracellular surface (Donoghue et al., 2000; Tipnis et al., 2000) and the ACE2 protein is encoded by the *ACE2* gene located on chromosome Xp22.

ACE2 is a monocarboxypeptidase, and *in vitro* can catalyze cleavage of the C-terminal residue of the peptides Ang I, des-Arg-bradykinin, neurotensin 1–13, and kinetensin (Donoghue et al., 2000), as well as catalyzing the hydrolysis of the C-terminal residue of Ang II (Tipnis et al., 2000). ACE2 can hydrolyze apelin-13 and dynorphin A 1–13 with high activity (Vickers et al., 2002), but its most important biological effect is to degrade Ang II to Ang 1-7, which it does with a catalytic efficiency that is 400-fold higher with Ang II as a substrate than with Ang I (Figure 1). Thus, ACE2 may limit the vasoconstrictor action of Ang II through its degradation, as well as counteracting the actions of Ang II through the formation of Ang 1-7 which is reported to have vasodilatory and anti-fibrotic actions (Tallant and Clark, 2003) at the Ang 1-7 or *mas* receptor (Santos et al., 2003). Ang 1-7 has other actions of cardiovascular relevance including natriuresis, diuresis, inhibition of cell growth and anti-atherosclerotic effects; the reader is referred to a recent review for a detailed description of the actions of Ang 1-7 (Santos, 2014).

This review will discuss the evidence regarding ACE2 in experimental models of primary and secondary arterial hypertension, the association between circulating ACE2 activity and blood pressure and hypertension in man, as well as genetic association studies that have investigated ACE2 polymorphisms with blood pressure and/or arterial hypertension.

## Experimental studies of ACE2

It is hypothesized that disruption of tissue ACE/ACE2 balance may result in changes in blood pressure, with increased ACE2 expression protecting against increased blood pressure, and ACE2 deficiency leading to increased blood pressure (Yagil and Yagil, 2003). Table 1 summarizes the experimental studies that have measured ACE2 gene, protein and/or activity, in either the heart, kidney and/or plasma, usually at one time point, and generally in animals with established hypertension, and compared results to a “normotensive” control.

**Table 1:** **ACE2 and experimental hypertension**.

**Aetiology**	**Model/Strain of hypertension**	**Control**	**ACE2 in hypertensive vs. control group**	**References**
**PRIMARY HYPERTENSION**				
Genetic	SHR	WKY	↓ Cardiac ACE2 mRNA, protein and activity	Zhong et al., 2004; Diez-Freire et al., 2006; Ferreira et al., 2011; Tan et al., 2011; Yang et al., 2013
			↓ Kidney ACE2 mRNA, protein and activity	Crackower et al., 2002; Zhong et al., 2004; Tikellis et al., 2006; Tan et al., 2011
			↓ ACE2 protein in rostral ventrolateral medulla	Yamazato et al., 2007
	SHR + 8% salt diet	SHR + 1% salt diet	↓ LV ACE2 mRNA and protein	Varagic et al., 2010
			↔ LV ACE2 mRNA and activity	Varagic et al., 2012
	Stroke-prone SHR	WKY	↓ Kidney ACE2 mRNA and protein	Crackower et al., 2002
			↔ kidney (cortical and medullary) ACE2 mRNA, and activity	Kamilic et al., 2010
	Sabra salt-sensitive rat (SBH/y) + normal chow	Sabra salt-resistant (SBN/y) + normal chow	↓ Kidney ACE2 mRNA and protein	Crackower et al., 2002
	SBH/y + DOCA-salt	SBN/y + DOCA-salt	↓ Kidney ACE2 mRNA and protein	Crackower et al., 2002
	SBH/y + 8% salt diet	SBH/y + normal chow	↔ Cardiac ACE2 protein	Landau et al., 2008
	Dahl salt-sensitive	Dahl salt-resistant	↓ Cardiac ACE2 mRNA and protein	Takeda et al., 2007
	**Transgenic models**			
	TGR(mREN2)27	SD	↔ Kidney (cortical and medullar) ACE2 mRNA, and kidney ACE2 activity	Kamilic et al., 2010
	mREN(2). Lewis	Lewis rats	↔ Cardiac ACE2 activity, ↓ kidney cortical ACE2 activity	Pendergrass et al., 2008
	TG(hRen + hAGT)	Non-TG mice	↔ Kidney ACE2 activity, ↑ brain ACE2 activity	Xia et al., 2009
**SECONDARY HYPERTENSION**				
Endocrine	DOCA + 1% salt in water + uninephrectomy in SD	Uninephrectomised rat (No DOCA + water)	↔ Plasma ACE2 activity	Ocaranza et al., 2011
			↔ LV ACE2 mRNA	Santiago et al., 2010
	DOCA + 1% salt in water + uninephrectomy in mice	Uninephrectomised mice (no DOCA + water)	↓ ACE2 protein and activity in hypothalamus	Xia et al., 2013
			↑ ACE2 activity in cerebrospinal fluid	
	Ang II infusion in SD rat	Vehicle infusion	↑ Cardiac ACE2 mRNA and protein, ↔ kidney ACE2 mRNA and protein	Meng et al., 2014
			↓ Kidney (cortical and medullary) ACE2 protein	Prieto et al., 2011
			↓ ACE2 mRNA and protein in paraventricular nucleus	Sriramula et al., 2011
	Ang II infusion in C57BL/6 mice	Vehicle infusion	↑ Plasma ACE2 activity, ↑ cardiac ACE2 mRNA, ↓cardiac ACE2 protein and activity	Patel et al., 2013a
			↓ Kidney ACE2 protein	Zhong et al., 2011
Renal	Goldblatt hypertension in SD	Sham operation	↓ Kidney ACE2 mRNA and protein	Prieto et al., 2011; Bai et al., 2013
	Goldblatt hypertension in HanSD	Sham operation	↑ Kidney ACE2 activity	Burgelova et al., 2009
	Subtotal nephrectomy	Sham operation	↑ Plasma ACE2 activity	Burchill et al., 2008
	(Acute kidney failure) in SD		↑ LV ACE2 mRNA, protein and activity	Burchill et al., 2008; Velkoska et al., 2011
			↓ Kidney cortical ACE2 mRNA, ↓ kidney (cortical and medullary) ACE2 activity	Velkoska et al., 2010
	Subtotal nephrectomy (chronic kidney failure) in SD	Sham operation	↔ Plasma ACE2 activity, ↔ LV ACE2 mRNA and protein	Burrell et al., 2012
			↓ Kidney (cortical and medullary) ACE2 mRNA and activity	Dilauro et al., 2010; Burrell et al., 2012

↑ Increased ACE2; ↓ decreased ACE2; ↔ no change in ACE2. Agt, angiotensinogen; Ang II, angiotensin II; DOCA, deoxycorticosterone; HanSD, Hannover-Spague Dawley rat; h, human; LV, left ventricle; Ren, renin; SBH, Saber Salt sensitive rat; SBN, Saber Salt resistant rat; SD, Sprague Dawley rat; SHR, spontaneously hypertensive rat; TG, transgenic; TGR(mREN2)27, transgenic renin hypertensive model; WKY, Wistar Kyoto rat.

## ACE2 and primary hypertension

There are several lines of evidence that support a role for ACE2 in the development of primary experimental hypertension. Firstly, the gene for ACE2 maps to a defined quantitative trait locus on the X chromosome, previously identified as a quantitative locus for blood pressure (Crackower et al., 2002), in three strains of genetically hypertensive rats the Sabra salt-sensitive rat, spontaneously hypertensive rat (SHR) and the stroke-prone SHR. ACE2 may play a role in the pathogenesis of genetic hypertension. In a study that examined ACE2 expression in the kidneys of SHR and normotensive Wistar-Kyoto (WKY) rats at birth, 6 weeks and adulthood (Tikellis et al., 2006), kidney ACE2 expression fell with the onset of hypertension in SHR compared to WKY, and remained reduced in the adult SHR kidney (Tikellis et al., 2006). Similarly, although cardiac ACE2 activity was not significantly different between 10 days old SHR and WKY rats, by 3 months cardiac ACE2 activity was reduced in SHR compared to age-matched WKY rats (Diez-Freire et al., 2006).

In adult rats with established hypertension, there was an association between reduced kidney ACE2 and blood pressure in SHR and in stroke-prone SHR, compared to levels in normotensive rats (Crackower et al., 2002). Reduced cardiac ACE2 expression (Zhong et al., 2004; Ferreira et al., 2011; Tan et al., 2011; Yang et al., 2013) and activity (Diez-Freire et al., 2006) has been reported in SHR compared to WKY rats, in Dahl salt-sensitive rats compared to salt-resistant rats (Takeda et al., 2007), and in salt-loaded SHR compared to SHR fed a low salt diet (Varagic et al., 2010). A subsequent study showed no change in cardiac ACE2 gene and activity in salt-loaded SHR (Varagic et al., 2012), and similarly in Sabra salt-sensitive rats, salt loading did not change cardiac ACE2 protein compared to salt-resistant Sabra rats on a normal diet (Landau et al., 2008).

The results with regard to regulation of tissue ACE2 in transgenic models of hypertension are mixed. Transgenic overexpression of renin in Sprague Dawley (SD) rats did not change kidney ACE2 expression and activity (Kamilic et al., 2010), but in transgenic Lewis rats, kidney cortical ACE2 was reduced with no change in cardiac ACE2 (Pendergrass et al., 2008). Similarly, in transgenic mice overexpressing human renin and angiotensinogen, no change in ACE2 activity was observed in the kidney, however this study reported elevated ACE2 activity in the brain of transgenic mice compared to non-transgenic mice (Xia et al., 2009). The disparity in observed changes highlights the importance of the strain and model used when examining the role of ACE2 in hypertension.

## ACE2 and secondary hypertension

Although a potentially reversible cause of hypertension can be identified in less than 10% of patients with hypertension, the overall high prevalence of hypertension means that secondary forms can affect millions of patients worldwide. Dysregulation of ACE2 has been reported in experimental models of secondary hypertension, such as endocrine hypertension [deoxycorticosterone (DOCA)-salt, Ang II infusion], renal failure (subtotal nephrectomy), and renal artery stenosis (Goldblatt hypertension).

In DOCA-salt hypertension in the rat, plasma ACE2 activity (Ocaranza et al., 2011) and left ventricular ACE2 gene expression (Santiago et al., 2010) were unchanged compared to uninephrectomised rats with no DOCA or high salt intake. Hypertension secondary to short term Ang II infusion (2 weeks) in SD rats resulted in decreased kidney cortical and medullary ACE2 protein (Prieto et al., 2011), although chronic Ang II infusion (4 weeks) in SD rats increased cardiac ACE2 expression with no change in kidney ACE2 (Meng et al., 2014). In mice, a 2 week Ang II infusion increased plasma ACE2 activity and cardiac ACE2 gene expression (Patel et al., 2013a), but reduced cardiac ACE2 protein and activity (Patel et al., 2013a) and kidney protein (Zhong et al., 2011).

In the Goldblatt (2-kidney, 1-clip) hypertensive SD rat model with kidney impairment, kidney ACE2 gene and protein expression were reduced in both clipped and non-clipped kidneys (Prieto et al., 2011; Bai et al., 2013), while renal ACE was significantly elevated, resulting in increased Ang II and reduced Ang 1-7 levels (Prieto et al., 2011). However, others have shown that kidney ACE2 and Ang 1-7 levels are increased in Goldblatt hypertension in Hannover SD rats without kidney impairment (Burgelova et al., 2009). In a short-term kidney failure model of hypertension induced by subtotal nephrectomy (STNx), we reported organ specific regulation of ACE2 with increased plasma and cardiac ACE2 (Burchill et al., 2008; Velkoska et al., 2011), and decreased kidney ACE2 (Velkoska et al., 2010). These data suggest that during the early stages of kidney impairment, activation of cardiac ACE2 may protect the heart against the adverse effects of an activated RAS, whilst depletion of kidney ACE2 contributes to kidney injury. Furthermore, in a long-term model of chronic kidney failure induced hypertension, we reported a persistent reduction in renal ACE2, but the early elevation in cardiac ACE2 was no longer evident, suggesting that this may contribute to ongoing cardiac dysfunction with renal disease progression (Burrell et al., 2012). In both short-term and chronic models of kidney failure induced hypertension, reduced kidney ACE2 was associated with increased blood pressure (Dilauro et al., 2010; Velkoska et al., 2010; Burrell et al., 2012).

## ACE2 and central control of blood pressure

ACE2 has also been identified in regions of the mouse brain involved in control of cardiovascular function (Doobay et al., 2007), with ACE2 gene deletion resulting in impaired baroreflex sensitivity and autonomic function (Xu et al., 2011), thus supporting a potential role of ACE2 in central blood pressure regulation. ACE2 protein expression was reduced in the rostral ventrolateral medulla (RVLM) of SHR compared to WKY rats, and overexpression of ACE2 in the RVLM decreased blood pressure in SHR but not WKY rats (Yamazato et al., 2007). Similarly reduced ACE2 activity in the brain stem was observed in genetically hypertensive mice (Xia et al., 2009), while specific ACE2 overexpression in the brain restored baroreflex function and contributed to the reduction of blood pressure in these mice (Xia et al., 2009). In Ang II induced hypertension, reduced ACE2 gene and protein expression in the periventricular nucleus (PVN) was reported, and adenoviral injection directly in the PVN reduced blood pressure (Sriramula et al., 2011). A recent study in DOCA-salt hypertensive mice, demonstrated that low-renin hypertension was associated with reduced ACE2 expression and activity in the hypothalamus and increased ACE2 activity in the cerebrospinal fluid, as a result of increased shedding of ACE2 from the tissue membrane by ADAM17 (a disintegrin and metalloproteinase) (Xia et al., 2013). Furthermore, knockdown of ADAM17 prevented the reduction of ACE2 in the brain, which was associated with a blunting of DOCA-salt hypertension (Xia et al., 2013). This data suggests that central ACE2 shedding by ADAM17 can also contribute to the development of hypertension.

## Effects of ACE2 inhibition/deletion and overexpression on blood pressure

The development of ACE2 inhibitors (Dales et al., 2002) and approaches to enhance ACE2 including viral transfection, recombinant ACE2 and ACE2 activators (Hernandez Prada et al., 2008; Kulemina and Ostrov, 2011), have helped to clarify whether ACE2 deficiency has a pathophysiological role in the onset of hypertension or is simply a consequence of elevated blood pressure. Treatment with the ACE2 inhibitor MLN-4760 had no effect on blood pressure in hypertensive mRen2 transgenic rats (Trask et al., 2010), nor in wild type mice (Tikellis et al., 2008). In mice with genetic deletion of ACE2 on the C57BL/6 background, ACE2 deficiency was associated with only a modest increase in blood pressure (Gurley et al., 2006; Tikellis et al., 2012), but enhanced susceptibility to Ang II-induced hypertension (Gurley et al., 2006). Other studies have shown no change in blood pressure in ACE2 knock-out (KO) mice compared to control C57BL/6 (Tikellis et al., 2008; Shiota et al., 2010), but once diabetes was induced in ACE2 KO mice, hypertension did develop (Tikellis et al., 2008). This suggests that ACE2 plays a compensatory role in the setting of disease, but not in normal physiology. This notion is supported by results from ACE2 overexpression studies. For example, cardiac ACE2 gene transfer reduced blood pressure and attenuated cardiac hypertrophy and fibrosis in SHR, but had no effect in normotensive WKY rats (Diez-Freire et al., 2006), and intra-cardiac ACE2 overexpression protected from angiotensin II-induced cardiac hypertrophy and fibrosis, but had no effect in vehicle infused rats (Huentelman et al., 2005). In addition, transgenic ACE2 overexpression in vessels of stroke-prone SHR reduced blood pressure and improved endothelial function (Rentzsch et al., 2008).

## Effects of enhancing ACE2 activity in experimental models of hypertension

It has been suggested that enhancing ACE2 activity may be a useful therapeutic approach in the management of high blood pressure. Current approaches to increase ACE2 activity include compounds that can activate ACE2 such as xanthenone (XNT) (Hernandez Prada et al., 2008) and all-trans retinoic acid (atRA) (Zhong et al., 2004). The compound atRA increased cardiac and kidney levels of ACE2 in SHR, reduced blood pressure and attenuated myocardial damage (Zhong et al., 2004). *In vitro* studies show that the ACE2 activator, XNT shifts the conformational equilibrium of ACE2 to increase its activity (Hernandez Prada et al., 2008), and acute administration in SHR reduced blood pressure, while chronic infusion reversed cardiac and renal fibrosis (Hernandez Prada et al., 2008). XNT infusion increased cardiac gene and protein ACE2 levels in SHR, which was associated with increased Ang 1-7 production and inhibition of ERK signaling (Ferreira et al., 2011). Central administration of XNT reduced acute air jet stress-induced increases in blood pressure (Martins Lima et al., 2013). By contrast, a recent study reported that XNT mediates its effects without activation of ACE2 (Haber et al., 2014). In a model of Ang II–induced acute hypertension, reduction in blood pressure was markedly enhanced by XNT with no effect on plasma nor kidney ACE2 activity (Haber et al., 2014). As the blood pressure-lowering effects of XNT were seen in both wild-type and ACE2 KO mice, this study concludes that the effects of XNT cannot be due to the activation of ACE2 (Haber et al., 2014). Further studies are needed to determine the precise mechanism of the antihypertensive and antifibrotic benefits of XNT.

A more direct approach to increase ACE2 is through recombinant ACE2. Recombinant human ACE2 (rhACE2) treatment partially reduced the pressor effect of an Ang II infusion without affecting basal systolic blood pressure in vehicle-treated mice (Zhong et al., 2010). Administration of rhACE2 also improved Ang II induced cardiac remodeling and dysfunction with attenuation of signaling pathways relevant for hypertrophy and fibrosis (Zhong et al., 2010). Both rhACE2 (Wysocki et al., 2010) and mouse recombinant ACE2 (Ye et al., 2012) attenuated Ang II induced hypertension in mice, with no effect on blood pressure in normotensive mice (Wysocki et al., 2010). However the initial increase in serum ACE2 observed in mice infused with rhACE2 was not sustained long-term due to the development of antibodies (Wysocki et al., 2010). The Ang II induced pressor effect in WKY rats was blunted with co-administration of rhACE2 (Lo et al., 2013), while in SHR 2-weeks of rhACE2 partially attenuated the blood pressure in association with reduced plasma Ang II levels and increased Ang 1-7 (Lo et al., 2013). Furthermore, lowering plasma and renal cortex levels of Ang II with rhACE2 over 4 weeks was associated with reduced blood pressure and slower progression of diabetic nephropathy in diabetic Akita mice (Oudit et al., 2010). A recent study examined the effect of intravenous administration of rhACE2 in healthy human subjects and found that a single rhACE2 dose of 100–1200 μg/kg was well tolerated and resulted in a dose-dependent increase in plasma ACE2, with no change in blood pressure or heart rate (Haschke et al., 2013). No antibodies to rhACE2 were detected 7, 14, and 28 days after the last dose of rhACE2 (Haschke et al., 2013). Studies evaluating the efficacy of this approach to lower blood pressure are currently lacking in patients with hypertension.

## Clinical studies of circulating ACE2 and hypertension

ACE2 undergoes “shedding” from endothelial cells to release the catalytically active ectodomain into the circulation (Lambert et al., 2005; Patel et al., 2013b). This process involves the proteinase ADAM17 (Lambert et al., 2005, 2008), and results in soluble ACE2, that can be measured in human plasma (Rice et al., 2006; Lew et al., 2008; Freeman et al., 2011; Chong et al., 2012; Soro-Paavonen et al., 2012; Ortiz-Perez et al., 2013; Roberts et al., 2013; Uri et al., 2014) using an ACE2-specific quenched fluorescent substrate assay described by Vickers et al. (2002). There are few studies that have specifically investigated whether circulating ACE2 activity is a biomarker of CVD in man, or if it is associated with blood pressure or hypertension. The results from the available studies suggest that plasma ACE2 activity is low in normal healthy volunteers (Lew et al., 2008), but increased in patients with cardiovascular risk factors or CVD (Freeman et al., 2011; Chong et al., 2012).

The Leeds Family study measured plasma ACE2 activity levels in healthy subjects and family members (Rice et al., 2006). Plasma ACE2 activity was detectable in only 40/537 subjects, and these subjects had significantly higher systolic and diastolic blood pressure compared to those without detectable plasma ACE2 (Rice et al., 2006). This study also provided evidence of genetic influences on circulating ACE2 activity and estimated that up to 67% of the variability in circulating ACE2 levels was explained by hereditable factors (Rice et al., 2006). This conclusion was based on the observation that among the 40 subjects with detectable ACE2 activity, half had at least one other family member with detectable ACE2 activity (Rice et al., 2006).

A number of other studies suggest an association between ACE2 activity and blood pressure. In patients with type 1 diabetes (*n* = 859), serum ACE2 activity was significantly higher in male compared to female patients, and was positively correlated with systolic blood pressure in both males and females (Soro-Paavonen et al., 2012). In patients with chronic kidney disease (*n* = 59), hemodialysis patients (*n* = 100) and kidney transplant recipients (*n* = 80) (Roberts et al., 2013), male patients had significantly higher plasma ACE2 activity compared to female patients; in females, plasma ACE2 activity was significantly associated with post-hemodialysis systolic blood pressure with a 4% increase in ACE2 for every 1 mmHg increase in systolic blood pressure. A recent study (Uri et al., 2014) has also reported that serum ACE2 activity was higher in hypertensive patients (*n* = 239) compared to healthy individuals (*n* = 45).

Others have reported no association between circulating ACE2 activity and hypertension. For example, monocyte-derived macrophage ACE2 activity in untreated hypertensive patients was not different to levels in normotensive individuals (Keidar et al., 2007), and in patients with ST-elevation myocardial infarction (*n* = 95), there was no association between ACE2 activity and hypertension (Ortiz-Perez et al., 2013). Whilst the studies by Soro-Paavonen et al. (2012) and Roberts et al. (2013) show that plasma ACE2 activity varies according to gender, most published ACE2 activity studies have not taken gender into account in their analysis.

## ACE2 gene association studies and hypertension

There have been 17 studies since 2004 (summarized in Table 2) investigating *ACE2* single nucleotide polymorphisms (SNPs) with blood pressure and/or hypertension (Burrell et al., 2013). These studies have been mainly conducted in Chinese populations (Huang et al., 2006; Yi et al., 2006; Zhong et al., 2006; Fan et al., 2007, 2009; Niu et al., 2007; Zhao et al., 2010a,b) with only four in Caucasians (Benjafield et al., 2004; Lieb et al., 2006; Patel et al., 2012; Malard et al., 2013), one in Koreans (Song et al., 2011) and one in an Indian population (Patnaik et al., 2014). Two *ACE2* SNPs, rs2285666 and rs1978124 are among those most frequently investigated in association studies. In three Chinese studies, significant associations in women with the rs2285666 SNP with either hypertension or systolic or diastolic blood pressure were reported (Yi et al., 2006; Zhong et al., 2006; Niu et al., 2007). In two of these studies, higher blood pressure was associated with the A allele (Yi et al., 2006; Niu et al., 2007) but in the third study it was the G allele (Zhong et al., 2006). Another study investigated orthostatic blood pressure responses in 3630 Chinese Han subjects and found no association with the rs2285666 SNP (Fan et al., 2009).

**Table 2:** ***ACE2* gene associations with blood pressure and/or hypertension**.

**References**	**ACE2 variant**	**Population**	**Gender**	**Study design**	**Sample size (case:control)**	**Phenotype**	**Significant findings with blood pressure and/or hypertension**
Benjafield et al., 2004	rs1978124	Australian white	Both	Case-control	152:193	Hypertension	No
	rs2285666	Anglo-Celtic origin					
	rs879922						
	rs714205						
Lieb et al., 2006	rs2285666	German Caucasian	Both	Cross-sectional	1674	Blood pressure in Healthy subjects in the MONICA Augsburg echocardiographic substudy	No
	rs4646156						
	rs879922						
	rs4240157						
	rs233575						
Huang et al., 2006	rs1978124	Chinese population of Han ethnicity	Both	Case-control	494:484	Hypertension	No
	rs2285666						
Yi et al., 2006	rs2285666	Chinese populations of (a) Han ethnicity	Both	Case-control	(a) 198:131	Hypertension	Yes
		(b) Dongxiang ethnicity			(b) 120:102		
Zhong et al., 2006	rs2285666	Chinese population of Han ethnicity	Both	Cross-sectional	353	Subjects with metabolic syndrome	Yes
Niu et al., 2007	rs1978124	Chinese population of Han ethnicity	Both	Case-control	808:686	Hypertension	Yes
	rs2285666						
Fan et al., 2007	rs2106809	Chinese population of Han ethnicity	Both	Two studies (a) Case-control	(a) 973:969	Hypertension	Yes
	rs2285666			(b) Clinical trial randomized to 4 treatment groups	(b) 3408		
	rs4646155						
	rs879922						
Zhou and Yang, 2009	rs2285666	Chinese population of Han ethnicity	Both	Meta-analysis (5 case-control studies)	2528: 2024	Hypertension	No
Fan et al., 2009	rs2106809	Chinese population of Han ethnicity	Both	Case-control	3630:826	Orthostatic blood pressure in Hypertensive patients	No
	rs2285666						
Zhao et al., 2010a	rs4646174	Chinese population of Han ethnicity	Both	Intervention study in probands and their families (parents, siblings, spouses and offspring) participating in the GenSalt Study	1906	Blood pressure responses to dietary potassium intake	Yes
	rs879922						
	rs4646140						
Zhao et al., 2010b	rs1514283	Chinese population of Han ethnicity	Both	Intervention study in probands and their families (parents, siblings, spouses and offspring) participating in the GenSalt Study	1906	Blood pressure responses to dietary sodium intake	Yes
	rs1514282						
	rs2074192						
	rs714205						
	rs4646176						
	rs2285666						
Song et al., 2011	rs1514282	South Korean	Both	Cohort study	7551	Blood pressure	Yes
	rs1514283						
Patel et al., 2012	rs1978124	Australian Caucasians	Both	Cohort study	503	Type 2 diabetes	Yes
	rs2074192						
	rs4240157						
	rs4646156						
	rs4646188						
Lu et al., 2012	rs2285666	Mixed ethnicity of Chinese Han, Dongxiang, Li and Australian Anglo Celtic	Both	Meta-analysis (9 case-control and 1 cross-sectional study)	7251:3800	Hypertension	Yes
Huang et al., 2012	rs1514283	Chinese population of Han ethnicity	Both	Probands and their families (parents, siblings, spouses and offspring) participating in the GenSalt Study	1998	Blood pressure response to cold pressor test	Yes
	rs4646176						
	rs879922						
Malard et al., 2013	rs2074192	Adolescents from 3 ethnicities, analysis performed separately: French Canadian European descent Other (biological parents of Asian, Spanish, African countries)	Both	Cohort study	555	Blood pressure	Yes
	rs233575						
	rs2158083						
	rs1978124						
Patnaik et al., 2014	rs2106809	South Asian population from Odisha, India	Both	Case-control	246:274	Hypertension	Yes

Two meta-analyses of the rs2285666 SNP have been reported with conflicting results (Zhou and Yang, 2009; Lu et al., 2012). One study had a combined total of 2528 Chinese Han subjects with hypertension and 2024 ethnicity matched normotensive controls and reported no association with hypertension (Zhou and Yang, 2009). The other study, a meta-analysis of 7251 hypertensive patients, reported a significant association between the rs2285666 AA genotype with hypertension in females only (Lu et al., 2012). However, this meta-analysis included mixed ethnicities (Chinese Han, Chinese Dongxiang, Anglo-Celtic) and reported significant heterogeneity in males, suggesting that the A allele was associated with hypertension in the Chinese Han subjects only. In one Korean study, the *ACE2* SNPs rs1514282 and rs1514283 were found to be significantly associated with diastolic pressure but not with hypertension (Song et al., 2011). A recent study investigated the *ACE2* gene in 246 hypertensive subjects from Odisha, India, and reported that the SNP rs2106809 TT genotype in females and the T allele in males was associated with hypertension compared to normotensive controls (Patnaik et al., 2014). Further studies with *ACE2* SNPs in other Indian populations will need to be conducted.

The evidence of *ACE2* gene associations in Caucasians is also inconsistent. In a small case-control study conducted in Anglo-Celtic Australian subjects with hypertension (*n* = 152) or normotension (*n* = 193), four *ACE2* SNPs were not associated with hypertension (Benjafield et al., 2004). In another study of healthy subjects participating in the MONICA Augsburg echocardiographic substudy, four *ACE2* SNPs (rs4646156, rs879922, rs4240157, and rs233575) were investigated but none were associated with systolic blood pressure variation (Lieb et al., 2006). However, in our own study of 503 Australian Caucasians with type 2 diabetes (Patel et al., 2012), the prevalence of hypertension was significantly higher in both men and women and was associated with the G allele of the *ACE2* SNP rs4240157 (Patel et al., 2012).

In another analysis, three *ACE2* SNPs (rs1514283, rs4646176, and rs879922) were reported to be significantly associated with the diastolic blood pressure response to a cold pressor test in female Chinese subjects participating in the GenSalt study (Huang et al., 2012). One study in the Chinese population reported no association of *ACE2* SNPs rs2106809 and rs2285666 with orthostatic blood pressure dysregulation in 3630 untreated hypertensive patients compared to 826 normotensive subjects (Fan et al., 2009).

The association of *ACE2* SNPs with baseline blood pressure and blood pressure changes over 5 years was examined in 555 adolescents mainly of French Canadian or European descent (Malard et al., 2013). This study restricted the analyses to homozygous females. In European males the *ACE2* SNP rs2074192 minor A allele was associated with a lower baseline diastolic and systolic blood pressure after weight and height adjustment. The *ACE2* SNP rs233575 C allele was associated with higher baseline systolic and diastolic blood pressure in European males. In contrast, French Canadian females with the rs233575 T allele had a 4 mmHg higher baseline diastolic blood pressure compared to the SNPs C allele. With regards to *ACE2* SNPs associated with changes in blood pressure from the baseline visit, there were no significant associations with the four *ACE2* SNPs investigated in European or French Canadian males. However in females of European descent, the rs2074192 A allele was associated with a change in systolic blood pressure that was lower and the C allele of SNPs rs2333575 and rs2158083 were associated with a change in systolic blood pressure that was higher compared to the alternative allele (Malard et al., 2013). This study demonstrates that the association of the *ACE2* SNPs with blood pressure was influenced by both gender and ethnicity.

## ACE2 gene and responses to blood pressure treatment

A small number of dietary and pharmacological interventions in Chinese populations have also been conducted in relation to *ACE2* polymorphisms. The GenSalt study in Chinese Han subjects examined responses to variation in sodium and potassium intake (Zhao et al., 2010a). This study reported *ACE2* SNPs to be associated with the reduction in blood pressure in response to potassium while on a high salt intake in males only (Zhao et al., 2010a) and also with variations in sodium intake in males and females combined (Zhao et al., 2010b). One study investigated the blood pressure response to RAS blockade in Chinese Han subjects (Fan et al., 2007), and reported that the diastolic pressure response to ACE inhibition was 3.3 mmHg greater in women with the rs2106809 T allele after adjustments for age, pre-treatment blood pressure, body mass index and fasting blood glucose. There are no studies of *ACE2* gene associations with blood pressure treatment responses in Caucasian populations.

## Summary and future directions

The experimental studies clearly support a physiological and pathophysiological role for ACE2 in arterial hypertension, and data is also available that increasing/activating ACE2 has beneficial effects to lower blood pressure. As therapeutic strategies that block the RAS using ACE inhibitors or Ang II receptor blockers are first line therapy in hypertension, experimental studies are needed that combine ACE2 activators/recombinant ACE2 with RAS blockers to determine if this approach offers incremental benefits.

There are very few clinical studies that have specifically investigated whether circulating ACE2 activity is associated with blood pressure or hypertension. Plasma ACE2 activity levels are low in healthy individuals, and may or may not be increased in patients with hypertension. Males have higher circulating ACE2 activity than females, but few reports have analyzed data according to gender. There are 17 studies that have examined *ACE2* genetic associations with blood pressure and hypertension; the majority are in Chinese populations, but the results have been inconsistent. There are not yet any studies that combine genetic approaches with measurement of plasma ACE2 activity. Large carefully conducted clinical studies, designed with sufficient numbers and power to allow separate analyses of males and females are urgently needed in patients with hypertension to more precisely clarify the potential role of ACE2 in blood pressure variation and hypertension, and to uncover mechanisms that might offer novel treatment targets.

### Conflict of interest statement

The authors declare that the research was conducted in the absence of any commercial or financial relationships that could be construed as a potential conflict of interest.

## Abbreviations

ACE: angiotensin converting enzyme
ACE2: angiotensin converting enzyme 2
ADAM17: a disintegrin and metalloproteinase
Ang: angiotensin
AT_1_R: angiotensin type 1 receptor
AT_2_R: angiotensin type 2 receptor
atRA: all-trans retinoic acid
CV: cardiovascular
CVD: cardiovascular disease
DOCA: deoxycorticosterone
KO: knockout
PVN: periventricular nucleus
RAS: renin angiotensin system
rhACE2: recombinant human ACE2
RVLM: rostral ventrolateral medulla
SD: Sprague Dawley
SHR: spontaneously hypertensive rat
SNP: single nucleotide polymorphism
STNx: subtotal nephrectomy
WKY: Wistar-Kyoto
XNT: xanthenone.
